# Manganese Neurotoxicity: A Comprehensive Review of Pathophysiology and Inherited and Acquired Disorders

**DOI:** 10.3390/jox15020054

**Published:** 2025-04-04

**Authors:** Giuseppe Magro, Vincenzo Laterza, Federico Tosto, Angelo Torrente

**Affiliations:** 1 Department of Neuroscience, “Giovanni Paolo II” Hospital, Lamezia Terme, 88100 Catanzaro, Italy; 2 Department of Medical and Surgical Sciences, Institute of Neurology, Magna Graecia University, 88100 Catanzaro, Italy; 3 Department of Biomedicine, Neuroscience, and Advanced Diagnostics (BiND), University of Palermo, 90129 Palermo, Italy; angelo.torrente@unipa.it

**Keywords:** manganese, hypomanganesemia, hypermanganesemia, manganese neurotoxicity, manganese exposed workers, manganism, SLC39A14, SLC39A8, SLC30A10

## Abstract

Manganese (Mn) is an essential trace element and a cofactor for several key enzymes, such as mitochondrial superoxide dismutase. Consequently, it plays an important defense role against reactive oxygen species. Despite this, Mn chronic overexposure can result in a neurological disorder referred to as manganism, which shares some similarities with Parkinson’s disease. Mn levels seem regulated by many transporters responsible for its uptake and efflux. These transporters play an established role in many inherited disorders of Mn metabolism and neurotoxicity. Some inherited Mn metabolism disorders, caused by mutations of SLC30A10 and SLC39A14, assume crucial importance since earlier treatment results in a better prognosis. Physicians should be familiar with the clinical presentation of these disorders as the underlying cause of dystonia/parkinsonism and look for other accompanying features, such as liver disease and polycythemia, which are typically associated with SLC30A10 mutations. This review aims to highlight the currently known Mn transporters, Mn-related neurotoxicity, and its consequences, and it provides an overview of inherited and acquired disorders of Mn metabolism. Currently available treatments are also discussed, focusing on the most frequently encountered presentations.

## 1. Introduction

Manganese (Mn) is a trace element in the body that is found in good concentrations in food [1]; for instance, the content of Mn in human milk is 3–10 μg/L, while that of soy formula is 100-fold higher (i.e., 200–300 μg/L) [2]. As a result, acquired Mn deficiency has not been reported in the literature so far [3]. Consequently, Mn deficiency disorders are mainly inherited. Mn plays a critical role in numerous biological systems, such as cell survival, bone formation, metabolism, and the antioxidant system [3]. Mn is a key element for the catalytic function of multiple metalloenzymes and many metabolic processes [4]. Mn supports a variety of biochemical and physiological processes. It also plays a critical role in enzymatic reactions since it acts as a cofactor for many enzymes, such as Mn superoxide dismutase (MnSOD), which is a key element involved in protection from oxidative damage [5]. Moreover, Mn is involved in arginase activity in the urea cycle, facilitating the removal of ammonia from the body [6]. Mn influences brain function in many ways, especially being a crucial component in neurotransmitter synthesis, including dopamine, glutamate, and gamma-aminobutyric acid (GABA), which are key elements for maintaining neural signaling and survival [7]. Mn homeostasis disruption has been involved in numerous neurological diseases, such as Parkinson’s disease (PD), Alzheimer’s disease, amyotrophic lateral sclerosis (ALS), prion disease, and Huntington’s disease [8]. Growing evidence suggests increased Mn exposure can negatively affect cognition, behavior, and intellectual functions, particularly in the developing brain [9].

Mn toxicity can result from an impaired or not fully developed excretion system, transporter malfunction, or excessive exposure to high levels of Mn in the air, food, water, or total parenteral nutrition. Mn toxicity and overload in humans have been commonly described as manganism, an acquired condition that shares many similarities with Parkinsonism. This syndrome is characterized by bradykinesia, rigidity, and tremor [10,11] and it results from the basal ganglia being the primary site of accumulation [12]. Mn intoxication is common in people with a history of mining, welding, battery manufacturing, and use of fungicides [13,14,15]. Nonetheless, Mn toxicity and exposure are not limited to miners or welders. Water and foods containing high levels of Mn represent an increasingly recognized source of contamination for the general population, with important implications for the health systems [16]. Levels of Mn in the atmosphere may also be increased due to gasoline additives [17]. Manganism classically requires occupational exposure of 6 months to 2 years. Once developed, symptoms resulting from Mn toxicity usually persist even when the source of Mn exposure is eliminated [18]. Drug abuse has recently become a recognized cause of Mn toxicity since abusers of methcathinone may be exposed to Mn due to the use of potassium permanganate in the synthesis process [19]. Mn accumulation results in oxidative stress, mitochondrial dysfunction, and the generation of reactive oxygen species (ROS) [20,21], which lead to neuronal apoptosis and neuroinflammation [22,23]. The effect of maternal Mn exposure on perinatal health has been investigated, and a recent systematic review found no significant association between Mn exposure and gestational diabetes mellitus; nonetheless, other pregnancy complications (e.g., risk of preterm birth) still need further investigation [24]. An inverted U-shaped relationship has been observed between Mn levels in maternal whole blood and birth weight, and between Mn levels in umbilical cord blood and birth weight [25]. Other studies have found that low and high maternal Mn levels are associated with adverse health outcomes in newborns [26,27]. Table 1 summarizes adequate Mn intakes for different age groups.

Understanding the balance between Mn’s physiological and pathological roles is crucial for developing preventive and therapeutic strategies against Mn-related neurotoxicity. The objective of the present review is to provide pathophysiological evidence of Mn neurotoxicity and the resultant effects of its accumulation in the brain, discussing the influence of genetic and environmental conditions.

## 2. Methods and Research Output

The articles for the main search were sought from PubMed with a publication date up to 31 January 2025. We limited the research to English-language manuscripts. The following search terms were used: “manganese”, “hypomanganesemia”, “hypermanganesemia”, “manganese neurotoxicity”, “manganese-exposed workers”, “manganism”, “slc39a14”, “slc39a8”, and “slc30a10”. As the main inclusion criterion, the main focus of the articles should have been occupational and genetic Mn neurotoxicity. We mostly used case series and human studies to describe Mn genetic disorders. The relative search string (“slc39a14” OR “slc39a8” OR “slc30a10”) yielded 539 results. For the Mn metabolism section, we used previous reviews. The relative search string (“manganese” OR “hypomanganesemia” OR “hypermanganesemia” OR “manganese neuro-toxicity” OR “manganese exposed workers” OR “manganism”), filtered to include review studies only, yielded 2973 results. Four authors (GM, AT, FT, and VL) examined the outputs of the research. The first screening was based on title only, followed by abstract and full-text screening. We also included relevant cited literature from the retrieved records. The authors were allowed to look for more articles based on their knowledge. All records were screened based on their relevance to the study objective; 131 records were included after careful screening.

## 3. Mn Transportation to the Brain

Mn transportation usually happens via dedicated transporters [29]. Multiple transporters for Mn have been described, but not all are selective for Mn transportation [9,29]. Several transporters are involved in Mn transport in the brain across the blood–brain barrier (BBB). Among the carrier proteins involved, the most important ones are divalent metal transporter 1 (DMT1), zinc-interacting protein 8 (ZIP8, encoded by SLC39A8), ZIP10 (SLC30A10), and ZIP14 (SLC39A14), other carriers involved in Mn transport are also transferrin and transferrin receptor (TR), citrate transporter, and calcium channels. Mn can be transported into the brain in different forms, such as Mn2+, Mn-citrate, or Mn3+-transferrin. As of today, all those mechanisms are believed to play a part in Mn transportation into the brain, and Mn transportation cannot be attributed to a single mechanism [30].

### 3.1. DMT1

DMT1 belongs to the family of natural resistance-associated macrophage proteins (NRAMPs) [31], which is a family of proteins involved in the host defense against several kinds of infections [32,33]. It is highly expressed in the plasma membrane and in the mitochondria, and it plays a role in mitochondrial iron and Mn acquisition [34]. DMT1 gene mutations are associated with severe microcytic anemia and iron overload [35]. DMT1 is ubiquitously expressed, with the highest expression in the basal ganglia, especially in the caudate nucleus, the putamen, and the substantia nigra. This is why these are the preferred manganese accumulation sites in Mn neurotoxicity [36]. Mn exposure induced DMT1 expression in mouse models in the subventricular zone [37]. DMT1 facilitates Mn transport in its divalent state (Mn2+), and it is regulated by iron status: iron deficiency enhances Mn absorption and brain accumulation [1]. Besides ingestion, olfactory absorption also plays a role in Mn uptake in the brain via DMT1 [38].

### 3.2. ZIP8 and ZIP14

ZIP8 is a transmembrane protein expressed on the surface of brain capillaries; it usually transports various divalent ions, including Mn, and it is encoded by the SLC39A8 [30,39]. ZIP8 is another membrane transporter whose activity has been recognized to be involved in the inhalation route since it is highly expressed in the lungs [40]. Nonetheless, it is also expressed at the apical side of the choroid plexus papilloma cells, and as such, it favors the influx of Mn through the blood–brain barrier [41]. ZIP8 (SLC39A8) is also localized to the apical membrane of hepatocytes, where it seems to reclaim Mn from the bile, suggesting a role for Mn homeostasis [42]. Mn deficiency mediated by ZIP8 impairment has recently been linked to a high degree of intestinal inflammation, leading to inflammatory bowel disease [43].

ZIP14, on the other hand, mediates the cellular transportation of Mn, Zn, and Fe and is encoded by the SLC39A14 gene [44]. ZIP14 seems to downregulate Mn absorption; as such, an animal model lacking ZIP14 accumulated higher Mn levels in the brain compared to the wild type [45]. Its function is due to the location, more specifically concentrated on the basolateral side of the choroid plexus cells, contributing to Mn transport through these cells and the subsequent excretion [41]. These findings are also supported by high Mn CSF levels in patients carrying an SLC39A14 mutation [46]. ZIP14 mutations lead to Mn accumulation in early-onset parkinsonism dystonia patients, thus highlighting an important role of ZIP14 in Mn level regulation [41].

### 3.3. SLC30A10

SLC30A10 (ZIP10) is one of the 10 solute carrier family 30 transporters. Unlike the majority of the family members of this family, which mediate zinc transport, the SLC30A10 mediates Mn efflux [47]. Its role in Mn-related inherited disorders is well established. Mutation of the SLC30A10 results in Mn accumulation and parkinsonism with dystonia, polycythemia, and liver cirrhosis. This protein is highly expressed in the digestive tract and liver [48].

### 3.4. Other Transportation Mechanisms

Citrate transporters, such as Mn-citrate, seem to be one of the major forms of Mn transported to the brain [49]. Mn-citrate crosses the blood–brain barrier with a citrate transporter-dependent mechanism [50]. Mn can also enter the brain through ligand-gated and voltage-gated calcium channels, respectively expressed in the brain endothelial cells and dopaminergic neurons of the midbrain. This could explain the selective vulnerability of these neurons to Mn toxicity [51].

Transferrin and transferrin receptor (TR) also help with Mn transportation. Transferrin mediates iron transport, but it can also favor the influx of Mn in the brain [52], in its trivalent state (Mn3+). TR is expressed in neurons, microglia, and astrocytes, and it binds the Mn-transferring complex into the cells [53]. This mechanism seems to play a less important role compared to others.

Another protein involved in Mn transportation is Fpn, a transmembrane protein expressed in the plasma membrane that exports intracellular iron [54]. Fpn plays an important role in regulating Mn levels in the brain, as it also exports intracellular Mn into the extracellular space, thus protecting against Mn toxicity [55].

## 4. Mechanisms Involved with Mn Toxicity

Mn toxicity can lead to the dysfunction of numerous pathways, such as the alteration and inhibition of mitochondrial respiration, resulting in energy failure, oxidative stress, and excitotoxicity [56,57].

Oxidative stress is one of the most implicated mechanisms of Mn-induced neurotoxicity. Mn accumulation in the brain can lead to damage in the basal ganglia, which are particularly susceptible to oxidative injury due to high oxygen demand and consumption. Mn increased oxidative damage and the activation of caspase-3, DNA fragmentation, and cytochrome c release from the mitochondria, which resulted in DNA fragmentation in dopaminergic cells [58]. Oxidative stress, a crucial mechanism of Mn-induced toxicity, was demonstrated in attenuated Mn neurotoxicity induced by antioxidant compounds, which also ameliorated dopaminergic transmission in experimental settings [59]. Mn also seems to exacerbate dopamine-induced oxidative damage since dopamine (DA) can undergo oxidation, producing free radicals [60]. Impairment of the neuronal antioxidant system may also result from altered striatal concentration of glutathione and glutathione reductase and peroxidase activity [61]. Oxidative stress can also result in nucleic acid damage, higher levels of oxidized DNA products have been observed in the substantia nigra and CSF of PD patients [62].

Another important mechanism of Mn-induced neurotoxicity is neuro-inflammation, which seems to be enhanced by astrocytes since Mn accumulates mainly in these cells in the brain [63]. This is probably the result of the abundant expression of many Mn transporters; among these, DMT1 and mostly TRs expressed on the astrocytic surface bind to Mn-transferrin with a resulting concentration of Mn inside the cell that is up to 50-fold greater than that in neurons [23]. Astrocytes are more susceptible to Mn-induced neurotoxicity; this leads to the release of many cytotoxic substances, such as iNOS, TNF-alfa, IL-1beta, and IL-6, in addition to the activation of numerous pro-inflammatory genes [30,64,65].

Mn seems to interfere with multiple neurotransmission systems. Evidence suggests involvement in DA, glutamate, acetylcholine, and GABA neurotransmitters [30]. Interestingly, Mn toxicity seems to result in decreased levels of DA and its turnover [66], depleted DA stores [67], decreased DA release in the striatum of rats, and concomitantly reduced dopaminergic neurotransmission [68]. Mn induces dopaminergic neuronal injury by decreasing the expression of tyrosine hydroxylase (TH) [69], and as a result, Mn leads to aberrant DA neurotransmission [30]. Glutamate neurotransmission is also impaired by Mn excess in the brain due to overstimulation of postsynaptic glutamate receptors and excitotoxic neuronal death [70]. N-methyl-D-aspartate receptor (NMDAr) is dysregulated by an altered phosphorylation mechanism and its subunits’ impairment. Mn exposure reduced glutamate uptake by astrocytes while simultaneously lowering the expression of glutamate aspartate transporter 1 (GLAST) and glutamate transporter 1 (GLT-1), ultimately leading to elevated extracellular glutamate levels [71]. Mn induces neuronal injury by disrupting glutamate signaling, partly through the impaired regulation of the glutamate transporters GLT-1/GLAST and the NMDA receptor [30]. Other neurotransmitters have been implicated in Mn neurotoxicity too; among these, the cholinergic system seems to be affected by Mn excess. Mn also reduced the activity of choline acetyltransferase (ChAT), thereby lowering ACh production in striatal cholinergic terminals [72]. Mn primarily accumulates in the GABAergic neurons of the globus pallidus within the basal ganglia, although its effects on GABAergic neurotransmission are variable. Some studies have found that Mn disrupts GABA signaling by reducing GABA levels, which in turn increases seizure susceptibility in rats [30].

### Protein Aggregation

Protein aggregation is another significant Mn-induced neurotoxicity mechanism, as it contributes to the progression and development of neurodegeneration. Protein misfolding, trafficking, and degradation are all impaired mechanisms resulting from chronic Mn exposure [1,73,74,75]. Mn can directly interact with proteins, causing misfolding and aggregation. This is the case of alpha-synuclein, which is a hallmark of neurodegenerative disorders, such as PD [76,77]. Mn alters the structural conformation of alpha-synuclein, increasing its propensity to form oligomers. These neurotoxic aggregates impair synaptic function and induce mitochondrial dysfunction [78]. Many studies have shown how Mn-induced alpha-synuclein aggregates alter cellular components, disrupt axonal transport, and activate microglial neuroinflammation [79,80]. Many other proteins are altered by Mn excess; among these, tau and TDP-43 are well recognized. Mn seems to promote tau hyperphosphorylation, which is linked to the formation of neurofibrillary tangles observed in tauopathies, such as Alzheimer’s disease [81]. Also, TDP-43, which is implicated in ALS and frontotemporal dementia (FTD), seems to be induced to form aggregates by Mn excess [56,82,83]. The impairment of protein degradation pathways is another leading mechanism of the Mn-induced aggregation process, and autophagy and the proteasome system are involved. Moreover, Mn impairs autophagy by disrupting lysosomal function and inhibiting autophagosome–lysosome fusion, leading to the accumulation of protein aggregates [78,79].

## 5. Hereditary Disorders of Manganese Metabolism

The first hereditary disorder of Mn metabolism was reported in 2012 [84]. The discovery led to the group of hereditary Mn transporter defects, hypermanganesemia with dystonia (HMNDYT 1 and 2) disorders being described. Usually, genetic forms manifest with developmental delay, childhood onset of “cock-walking” gait, and dystonia with or without parkinsonism. Mutations in SLC30A10 lead to a syndrome of hypermanganesemia with dystonia, polycythemia, and chronic liver disease, referred to as hypermanganesemia with dystonia 1 (HMNDYT1) (OMIM#613280) [85]. In 2016, a comparable hereditary Mn transporter defect known as hypermanganesemia with dystonia 2 (HMNDYT2) (OMIM #617013) was reported, which can be differentiated from SLC30A10 deficiency by the lack of liver involvement and polycythemia. Mutations in SLC39A14 result in swiftly advancing dystonia accompanied by varying degrees of parkinsonism and other neurological symptoms, typically beginning in infancy or early childhood. Both types of inherited Mn transporter defects exhibit distinctive MRI brain characteristics, which are marked by hyperintensity on T1-weighted images in areas such as the globus pallidus and striatum, as well as in the white matter of the cerebrum and cerebellum, midbrain, dorsal pons, and medulla. Notably, the ventral pons is generally unaffected by these changes [45,84,85].

Table 2 highlights the clinical features of genetic and acquired Mn-overload syndromes.

### 5.1. HMNDYT1 SLC30A10 Deficiency

Hypermanganesemia with dystonia 1 (HMNDYT1), resulting from bi-allelic mutations in SLC30A10, was the initial inherited defect in Mn transporters identified [84,85]. The accumulation of Mn in the body due to systemic factors results in a unique syndrome characterized by hypermanganesemia, an increase in red blood cell count (polycythemia), movement disorders (dystonia), and chronic liver diseases, which can vary from asymptomatic fat accumulation in the liver (steatosis) to severe liver damage (cirrhosis) accompanied by liver failure, as well as a reduction in iron reserves. Urinary and blood Mn levels are significantly elevated, typically reported to be ten times higher than normal. When examining brain MRI scans, Mn deposition can be observed in the basal ganglia, especially in the globus pallidus and striatum. This is characterized by a marked hyperintensity on T1-weighted images and a corresponding hypointensity on T2-weighted images, indicating the presence of Mn in these areas. The white matter of the cerebrum and cerebellum, midbrain, dorsal pons, and medulla are also affected, while the ventral pons shows a characteristic sparing [84,85,86,87,88,89]. Clinically, most patients exhibit dystonia in early childhood. Dystonia in the lower limbs results in a distinct high-stepping gait, often referred to as the “cock walk gait”. The involvement of white matter may lead to spasticity and signs of pyramidal tract dysfunction [84]. From a histological perspective, significant neuronal loss in the globus pallidus and a vacuolated myelinopathy have been reported [90]. The brain is not the only organ where Mn deposits accumulate; the liver is the second most commonly involved organ that may present damage induced by Mn toxicity. Liver damage can range from mild (steatosis) to more severe (cirrhosis). Polycythemia has been observed in all patients and may occur before clinical symptoms appear. It has been proposed that Mn triggers the expression of the erythropoietin gene. Erythropoietin levels have been elevated in some affected individuals [85]. Given that Mn and iron (Fe) compete for similar transporters, it is not unexpected that individuals with mutations in the SLC30A10 gene exhibit depleted iron stores. These individuals also demonstrate an elevated total iron-binding capacity and a reduced level of ferritin [84,85]. Chelation therapy using EDTA-CaNa2 has proven to be effective for decreasing Mn accumulation, alleviating neurological symptoms, and halting the progression of liver disease. In many instances, the process of Mn chelation results in the resolution of polycythemia, the normalization of iron parameters, and the stabilization of blood Mn levels. Nevertheless, it is important to note that blood Mn levels frequently do not return to normal and may remain elevated [88,91,92]. A successful decrease in Mn levels can be observed through brain MRI, which shows a decrease in T1 hyperintensity. EDTA-CaNa2 is administered intravenously throughout 5 to 8 days every 4 weeks. It is essential to closely monitor calcium and other trace metal levels, including zinc (Zn), copper (Cu), and selenium (Se), to prevent any negative side effects [93]. Although chelation using EDTA-CaNa2 is effective, the requirement for intravenous administration renders the treatment less practical. Some case reports indicate that 2,3-dimercaptosuccinic acid and d-penicillamine offer effective oral alternatives [87,94]. It is unclear whether this alternative treatment can slow disease progression as effectively as EDTA-CaNa2.

### 5.2. HMNDYT2 SLC39A14 Deficiency

In 2016, bi-allelic mutations in *SLC39A14* were identified in individuals with features of Mn neurotoxicity, such as rapidly progressing dystonia, variable parkinsonism, and T1 hyperintensity in the globus pallidus observed on brain MRI [45]. Individuals with HMNDYT2 exhibited hypermanganesemia but lacked systemic features of Mn overload, such as liver disease or polycythemia. Blood levels of Fe, Zn, and cadmium (Cd)—divalent metals transported by SLC39A14—are usually normal. Additionally, liver MRI findings are usually normal, indicating no hepatic Mn accumulation. Neurological symptoms in HMNDYT2 tend to manifest earlier than in HMNDYT1, with some individuals experiencing severe hypotonia and dystonia within their first year of life [45,91]. MRI images show no difference from those of HMNDYT1.

The cornerstone of treatment involves chelation therapy using intravenous disodium calcium edetate (EDTA-CaNa2) and iron supplementation. This treatment algorithm is consolidated and shows greater response in HMNDYT1 [84,85,86,93]. Chelation therapy using EDTA-CaNa2 has been tried with variable degrees of success in HMNDYT2 [45]. Although there is evidence of Mn mobilization, indicated by increased urinary excretion and a reduction in blood Mn levels, the neurological symptoms in these individuals did not show significant improvement [45,46,95]. Two oral chelating agents, 2,3-dimercaptosuccinic acid and d-penicillamine, were tested in one patient but were ineffective, failing to increase urinary Mn excretion [95].

### 5.3. Manganese Disorders Associated with Low Levels of Mn

Conversely, mutations in SLC39A8, another transporter for Mn uptake, have been linked to lower Mn levels in the blood. A deficiency in Mn results in reduced activity of Mn-dependent enzymes, such as β-1,4-galactosyltransferase and MnSOD, which can cause dysglycosylation, known as congenital disorder of glycosylation type IIn (CDG2N), along with compromised mitochondrial function [96,97,98]. Individuals affected may show signs from infancy, including developmental delays, short stature, dwarfism, seizures, hypotonia, and dystonia. Oral treatment with Mn and galactose may offer potential strategies for management [99].

## 6. Acquired Disorders of Manganese Metabolism

Numerous studies have suggested a high brain vulnerability to Mn, especially during developmental phases. Exposure to increased Mn concentrations in drinking water correlates with neurological abnormalities in children and psychological impairment. These neurological abnormalities result in behavior and cognition impairment, as well as motor functions with tremors and coordination difficulties [100]. The most well-known entity of acquired Mn neurotoxicity is the so-called manganism in people who misuse drugs and especially exposed workers (such as miners). Occupational exposure to inhaled Mn compounds in the welding industry accounts for many reported cases [89,101].

Mn toxicity can also result from the misuse of intravenous ephedrone (or methcathinone) due to the potassium permanganate required for the drug’s synthesis, which can deposit in the brain, leading to manganism symptoms [102]. This parkinsonian syndrome was noted in methcathinone users in Russia and the Baltic states [103]. Methcathinone is a stimulant with euphoric effects; it is derived from a process of potassium permanganate oxidation for the intravenous formulation. One of the largest case series of 23 adults in Latvia reported the most frequent symptoms to be gait disturbance, which occurred after a mean of 5.8 years from methcathinone use; difficulty walking backward seemed to be a distinguishing feature. Tremors during rest were not a distinguishing feature, which is commonly observed during PD. Methcathinone patients had a symmetric motor disorder, fell frequently, walked on the balls of their feet (the typical “cock walk gait”), had profoundly soft speech, and did not respond to treatment with levodopa [19,104,105].

Acquired hepatocerebral degeneration patients with advanced cirrhosis present an excess of brain Mn accumulation and associated clinical features of neurotoxicity [106]. Interestingly, in one study, T1 hyperintensity of the globus pallidum was reported in 26 out of 90 cirrhotic patients on the liver transplant waiting list [107].

Another well-known cause of excessive Mn accumulation is total parenteral nutrition, especially in neonates, which can have critical negative consequences since parenteral nutrition bypasses the little-developed homeostatic mechanism of the gut and liver [3,13,102]. Toxicity related to Mn exposure was noted as little as 15 days after parenteral nutrition. The Mn dosage, often influenced by its content in commercially available products, may frequently surpass the limits recommended by clinical guidelines and should be restricted to 55 µg/day [108].

Despite the route, Mn seems to accumulate in the brain regions with dopaminergic pathways (mainly in the basal ganglia and mostly in the globus pallidum), potentially leading to dopaminergic dysfunction. MRI imaging for Mn encephalopathy typically reveals symmetrical hyperintensities on T1-weighted images, predominantly in the basal ganglia. These changes are most evident in the globus pallidus and cerebellum. Changes in the hypothalamus and midbrain are less common [109,110,111]. Symptoms of Mn encephalopathy are often permanent, and symptomatic treatments, such as the use of anti-Parkinson drugs, typically show limited effectiveness [105,112].

Mn role has also been investigated in autism spectrum disorder (ASD): an increasing number of studies suggest an inverse relationship between systemic Mn levels and ASD. Conversely, the majority of findings report elevated systemic Mn levels in individuals with attention-deficit/hyperactivity disorder [113].

**Figure 1:**
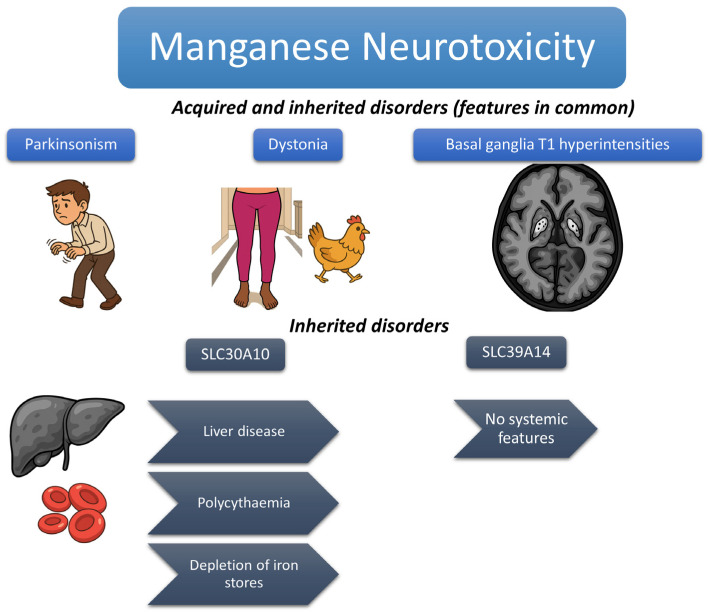
The figure shows the typical features of acquired and inherited Mn metabolism disorders. Parkinsonism and basal ganglia T1 hyperintensities are shared features, as is the classical “cock-walk” gait (a particular form of dystonic gait in which patients walk on their toes with a high-stepping gait that is more classically reported in inherited forms but described in both). Other distinguishing features of inherited forms, typically associated with SLC30A10 mutations, are highlighted.

Figure 1 summarizes distinguishing features between acquired and inherited Mn disorders.

## 7. Distinguishing Features from Parkinson’s Disease

Mn toxicity shares many similarities and clinical features with PD, such as bradykinesia, rigidity, tremors, and postural instability. Nonetheless, many differences exist in the clinical presentation, underlying mechanisms, and treatment responses. Unlike PD, which is usually associated with dopaminergic neuron impairment in the substantia nigra, Mn toxicity usually leads to excessive Mn accumulation in the basal ganglia, especially in the globus pallidus, without the presence of Lewy bodies [114,115]. Magnetic resonance of Mn-exposed individuals typically shows T1 diffuse hyper-intensities, especially in the globus pallidus, which is not seen in PD [10,101,116]. An important clinical distinction is the lack of resting tremors in manganism, usually present in PD. Mn neuro-toxicity is usually more commonly associated with action tremors and dystonia [10,117]. Cognitive symptoms and neuropsychiatric impairment are usually earlier features compared to PD [9,118,119]. Another main difference is the treatment response to levodopa, manganism does not respond to levodopa given the absence of primary involvement of the substantia nigra. Mn seems to primarily disrupt dopaminergic transmission through interference with dopamine release, synthesis, and reuptake [7,12,52,120]. On the other hand, manganism tends to stabilize upon removal from Mn exposure, whilst the progression of the disease is well recognized in PD [19].

## 8. Therapeutic Options

Chelation therapy remains a cornerstone for treating Mn toxicity to reduce its neurotoxic effects. Among the most used therapeutic agents, chelation therapy with disodium edetate has been administered to patients with hypermanganesemia disorders to reduce Mn levels, but its effectiveness has been inconsistent [45,46,87,88,89]. A motor improvement has been reported in some patients, whilst others do not respond to the therapy at all. Some patients with monogenic inherited causes of hypermanganesemia have shown clinical improvement, including decreased Mn levels, stabilized gait, reduced bradykinesia, and improved dystonia, following chelation therapy [45,84,86,88]. Improvement with chelation therapy in genetic Mn disorders is not always to be expected. Clinical response may depend on many variables, such as age and time of treatment. Acquired causes of Mn do not always respond to chelation treatment, and symptoms may continue even many years after exposure cessation [12]. Moreover, chelation intravenous therapy increases the risk of side effects and requires close monitoring of other essential trace metals for reduced absorption. Ethylenediaminetetraacetic acid (EDTA) and calcium disodium EDTA are the most extensively studied chelators for Mn toxicity, demonstrating effectiveness in reducing blood Mn levels in cases of both occupational and environmental exposure [1]. Recent studies have explored using newer chelating agents, like deferoxamine (DFO) and deferasirox (DFX), which were initially developed for treating iron overload. These agents have shown promise in mobilizing Mn from tissues and reducing neuroinflammatory markers [76,121]. Given the intravenous administration of calcium disodium EDTA and its side effects and low availability, other strategies are being investigated. Among those, penicillamine has reported variable responses for inherited disorders, such as hypermanganesemia with dystonia, polycythemia, and cirrhosis [92,94,122,123]. Interestingly, iron supplementation may lower Mn concentration by competing for the same transport mechanism. Animal studies have demonstrated the neuroprotective effects of DFO, showing its ability to reduce oxidative stress and restore mitochondrial function in cases of Mn-induced neurotoxicity [56,80].

The effectiveness of chelation therapy depends on the duration and severity of Mn exposure. Early intervention is essential, as extended exposure may cause irreversible neurodegeneration, reducing the efficacy of chelating agents. Antioxidants are gaining attention as a potential therapy for manganese-induced neurotoxicity by targeting oxidative stress, a key factor in Mn-related neuronal damage. Recent research showed that antioxidants, like N-acetylcysteine (NAC), can effectively decrease ROS and enhance mitochondrial function in cells and animal models exposed to Mn [1]. Many other antioxidants are being investigated, but at the moment, their use in clinical practice remains controversial. Mn supplementation appears to be an effective treatment for patients with inherited Mn deficiency, as shown in a study that reported significant improvement in motor function and neurological symptoms in two patients with SLC39A8 after Mn supplementation [99].

Dietary approaches include high-iron diets that suppress Mn absorption [124]. Also, adding calcium to human milk significantly decreases Mn absorption; a similar effect is observed with dietary phytate, which can reduce the absorption of many other minerals [125]. Another approach could be avoiding foods rich in Mn, such as grain products, tea, vegetables, and seafood, like mussels [28,126].

As for symptomatic treatment, Levodopa has been administered as a symptomatic treatment to some patients with hypermanganesemia, showing variable effectiveness in alleviating symptoms. Several cases of acquired hepatocerebral degeneration have also reported clinical improvement with levodopa therapy [127]. Welders exposed to Mn do not seem to respond to levodopa treatment, regardless of the dosage [128]. Patients with inherited Mn transport disorders have exhibited a variable clinical response to levodopa treatment [84,86,89].

## 9. Future Directions

Despite the progress in understanding the pathophysiology of Mn disorders, many questions remain unsolved. Among these, the timing of Mn deposition in the brain remains elusive, such as the fact that different brain regions seem to exhibit different elimination rates of Mn upon cessation of exposure [129]. A comparison is needed between dopamine terminals and dopamine release in individuals with PD and those with manganism. In cases of manganism, both mice and primates exhibit intact dopamine terminals but impaired dopamine release; in contrast, individuals with Parkinson’s disease show impairments in both dopamine terminals and release [114].

No well-established biomarkers exist for Mn-induced damage to the central nervous system yet. Although animal studies have provided valuable insights, these findings still need to be validated in humans [9]. Metabolomics offers promising potential for identifying candidate markers of Mn exposure, uncovering disrupted biological pathways, and detecting early signs of Mn toxicity. However, its application in exposure assessment faces several challenges, mainly due to the natural variability of small-molecule metabolites [130]. It is also essential to understand the role of other metals in diagnosing manganism, especially since low iron levels are often observed in cases of hypermanganesemia. Recent studies have shown that Mn exposure affects the transcriptional regulation of key pathways, including those related to oxidative stress, through Mn-induced modulation of sirtuin and Keap1-Nrf2 signaling. Also, a significant role of autophagy as a protective mechanism against Mn neurotoxicity has been described [131]. Many gaps related to Mn toxicity still remain to be filled in. The present review discussed the pathological implications of Mn transportation disruption in environmental and genetic conditions. The latter provide a good disease model to enhance our comprehension of Mn toxicity.

## 10. Conclusions

Although progress has been made in understanding Mn neurotoxicity, there are still significant challenges in early diagnosis, effective treatment, and long-term management of these conditions. Further research is needed to identify reliable biomarkers, develop targeted therapies, and conduct longitudinal studies to gain deeper insights into the long-term effects of Mn neurotoxicity.

## Figures and Tables

**Table 1:** Suggested Mn intakes per age and sex. Measurement units are in milligrams (mg).

Age	Male	Female	Pregnancy-Lactation
Birth to 6 months	0.003	0.003	
7–12 months	0.6	0.6	
1–3 years	1.2	1.2	
4–8 years	1.5	1.5	
9–13 years	1.9	1.6	
14–18 years	2.2	1.6	2.0
19–50 years	2.3	1.8	2.0
51+ years	2.3	1.8	

Adapted from Institute of Medicine (US) Panel on Micronutrients. Dietary reference intakes for vitamin A, vitamin K, arsenic, boron, chromium, copper, iodine, iron, manganese, molybdenum, nickel, silicon, vanadium, and zinc. Washington (DC): National Academies Press (US); 2001 [28].

**Table 2:** Summary of inherited and acquired causes of manganese (Mn) neurotoxicity. Legend: TPN: total parenteral nutrition.

Disorder	Etiology	Age of Onset	Clinical Features	Diagnostic Evaluation	Management
***Inherited***					
**SLC30A10 Deficiency**	Mutation in the *SLC30A10* gene impairs Mn efflux, resulting in systemic Mn accumulation	Childhood; often early onset	Dystonia, parkinsonism, gait abnormalities, polycythemia, and chronic liver dysfunction	Elevated blood/urine Mn; MRI shows symmetrical T1 hyperintensities in basal ganglia; confirmed by genetic testing	Mn chelation (e.g., EDTA-based regimens), dietary Mn restriction, and supportive therapies
**SLC39A14 Deficiency**	Mutation in the *SLC39A14* gene disrupts Mn uptake regulation, causing toxic accumulation	Childhood; early onset	Early-onset parkinsonism–dystonia, spasticity, and progressive neurodegeneration	Elevated Mn levels; characteristic MRI findings; genetic confirmation via mutation analysis	Chelation therapy and supportive management; treatment response may vary, so early intervention is critical
***Acquired***					
**Environmental Exposure**	Exposure to Mn through contaminated water, soil, or ambient air in industrial/urban areas	Variable; can affect both children (in high-exposure regions) and adults	Parkinsonian features, such as tremor, rigidity, and bradykinesia; in children, potential developmental delays or learning difficulties	Environmental exposure history; measurement of Mn in biological samples; MRI with basal ganglia T1 hyperintensities	Reduction of environmental exposure
**Iatrogenic Exposure (TPN-Related)**	Excess Mn is inadvertently administered via total parenteral nutrition (TPN)	Any age receiving long-term TPN	Neurological signs, including dystonia and parkinsonism-like symptoms	History of prolonged TPN use; elevated Mn levels; exclusion of alternative causes	Adjusting TPN formulations to limit Mn content; discontinuation or modification of TPN regimens; symptomatic management
**Hepatic Dysfunction-Associated Accumulation**	In patients with chronic liver disease or cirrhosis, impaired biliary excretion leads to Mn accumulation in the brain	Typically, adults with chronic liver disease	Neurological manifestations resembling parkinsonism; may contribute to features of hepatic encephalopathy	Liver function tests; elevated Mn levels; MRI revealing T1 hyperintensities in the basal ganglia	Management of the underlying liver disease, reduction of Mn intake, and supportive/symptomatic therapy

## Data Availability

No new data were created or analyzed in this study.
